# EQUIPTT: The Evaluation of the QUiPP app for Triage and Transfer protocol for a cluster randomised trial to evaluate the impact of the QUiPP app on inappropriate management for threatened preterm labour

**DOI:** 10.1186/s12884-019-2210-1

**Published:** 2019-02-13

**Authors:** Helena A. Watson, Naomi Carlisle, Katy Kuhrt, Rachel M. Tribe, Jenny Carter, Paul Seed, Andrew H. Shennan

**Affiliations:** Division of Women’s Health, Faculty of Life Sciences & Medicine, King’s College London, 10th Floor, North Wing, St Thomas’s Hospital Campus, London, SE1 7EH UK

**Keywords:** Preterm birth, Prediction, Fetal fibronectin, Cervical length, QUiPP app

## Abstract

**Background:**

Accurate diagnosis of preterm labour is needed to ensure correct management of those most at risk of preterm birth and to prevent the maternal and fetal risks incurred by unnecessary interventions given to the large majority of women, who do not deliver within a week of presentation. Intervention “just-in-case” results in many avoidable admissions, women being transferred out of their local hospital unnecessarily and most women receiving unwarranted drugs, such as steroids and tocolytics. It also precludes appropriate transfers for others as neonatal cots are blocked pre-emptively, resulting in more dangerous ex-utero transfers. We have developed the QUiPP App which is a clinical decision-making aid based on previous outcomes of women, quantitative fetal fibronectin (qfFN) values and cervical length. It is hypothesised that using the QUiPP app will reduce inappropriate admissions and transfers.

**Methods:**

A multi-site cluster randomised trial will evaluate whether the QUiPP app reduces inappropriate management for threatened preterm labour. The 13 participating centres will be randomly allocated to receive either intervention or control. If the QUiPP app calculates risk of delivery within 7 days to be is less than 5%, clinicians are advised that interventions may be withheld. Women’s experience of threatened preterm labour assessment will be explored using self-completed questionnaires, with a subset of participants being invited to semi-structured interview. A health economics analysis is also planned.

**Discussion:**

We hypothesise that the QUiPP app will improve identification of the most appropriate women for admission and transfer and ensure that therapies known to reduce risk of preterm neonatal morbidities are offered to those who need them. We will determine which women do not require these therapies, thereby reducing over-medicalisation and the associated maternal and fetal risks for these women. The findings will inform future national guidelines on threatened preterm labour. Beyond obstetrics, evaluating the impact of an app in an emergency setting, and our emphasis on balancing harms of over-treatment as well as under-treatment, make EQUIPTT a valuable contribution to translational medicine.

**Trial registration:**

The EQUIPTT trial was prospectively registered on 16th January 2018 with the ISRCTN registry (no. 17846337).

## Background

Women with symptoms of preterm labour have long posed a diagnostic challenge for clinicians concerned with managing the risks of preterm birth when the reality is that most women will not deliver imminently [1]. Various prediction methods are available to direct interventions that delay or ameliorate the consequences of preterm birth (e.g. in-utero transfer, tocolysis, antenatal corticosteroids). However these interventions have psychological, economic and clinical implications if given unnecessarily.

In light of recent UK guidance to treat all women in threatened preterm labour (TPTL) prior to 30 weeks [2], most of these women are likely to be admitted. However, the majority of women presenting with symptoms of preterm labour will not deliver in seven days [1].

## Risks associated with antenatal corticosteroids

Clinicians are familiar with steroid-induced glucose intolerance in the mother, necessitating insulin/dextrose infusions and prolonged admissions. However, fetal exposure to steroids has become the over-arching concern. There is a significant reduction in infant birthweight of those women exposed to antenatal corticosteroids who deliver more than seven days after the first dose, compared to those receiving no treatment (mean difference 147 g, 95% CI -291.97 to − 2.05 g) [3]. Infants exposed to steroids are at increased risk of neonatal hypoglycaemia (1.61 CI 1.38–1.87) and are significantly more likely to be in the lower quartile of academic ability (*p* = 0.01) (ARR 9.2–17.7% to 8.5%) [4]. There is biological plausibility supporting the latter finding given evidence of decreased brain growth in preterm and term infants exposed to antenatal corticosteroids in animal studies [5]. There is also evidence that fetal exposure to glucocorticoids modifies fetal hypothalamic-pituitary-adrenal function, which is associated with increased risk of cardiovascular and metabolic diseases in adulthood [6, 7]. Structural development of the brain may also be affected by steroid exposure, such as impaired cortical folding [8] and cortical thinning [9].

## Risks associated with unnecessary in-utero transfer

Appropriate antenatal in-utero transfer (as opposed to ex-utero) is essential to avoid the significant increases in neonatal morbidity and mortality associated with postnatal transport of preterm infants [10]. In some units a treat-all policy for threatened preterm labour would dramatically increase the number of such transfers. The implications of this far exceed the ambulance transfer costs: being transferred to another hospital unit (out of area) is associated with a significant stress and expense for the parents and family [11]. There is an immediate clinical burden of transfer arrangements, which involves a number of on-call obstetric, neonatal and midwifery teams and takes an average of 340 min to complete [12]. This does not include the return journey time of the transferring midwife. Paradoxically, unwarranted antenatal transfers may increase more dangerous postnatal transfers by impairing efficient management of neonatal cots (i.e. cots reserved for babies that do not actually deliver preterm). With these neonatal cots blocked, infants of women in true preterm labour are only able to be transferred postnatally.

## Maternal risks

Hospitalisation also increases the risks of venous thromboembolism, hospital-acquired infections and the psychological strain for the mother.

Our research group has developed the QUiPP App, which improves prediction of preterm labour by combining clinical parameters and calculates risks more precisely from continuous variables, to better assess risk [13–15]. It utilises quantitative fetal fibronectin (qfFN) (a protein released into the vagina at high concentrations during preterm labour), the use of which has been developed by our group [13–15]. In a prospective observational study of 300 women, we reported a negative predictive value (NPV) for qfFN at < 10 ng/mL of 98.2%, and a positive predictive value (PPV) for delivery < 34 weeks at a 200 ng/mL threshold of 37% [13]. The value is limited by these arbitrary thresholds, and we showed that improved prediction can be obtained by interrogating the data across the whole qfFN range i.e. better sensitivity and “rule out” at low levels and improved specificity and “rule in” at high levels. We combined qfFN and cervical length measurements (also as a continuous variable) to improve the prediction further in 1249 asymptomatic women [14]. We then created a predictive model from a prospective observational dataset of 382 women with TPTL to provide an individualised risk of delivering within 1/2/4 weeks and before 30/34/37 weeks [15]. The accuracy of the model was further confirmed using a validation set and performance reliability demonstrated by comparison of expected and observed sPTB rates (*p* > 0.05). Receiver operating characteristic areas in the validation set differed from the training set by amounts between − 0.04 and + 0.02 [15]. The model was subsequently updated on a larger dataset comprising 1032 women presenting with symptoms of TPTL, and this is the analysis that is incorporated into the latest version of the QUiPP app.

In terms of guiding management decisions, the majority of cases are assigned a very low-risk (< 1%) by QUiPP app, which clearly indicates a “no admit” strategy. In EQUIPTT, we provide a guidance threshold of 5% risk of delivery within seven days for intervention, based on our previous published work [16]. However, given the other multiple variables that contribute to this decision (such as gestational age), and our intention for the QUiPP app to be a tool to help clinicians make their own decisions, we have refrained from dogmatic recommendation based on absolute thresholds.

We also want to establish if use of the QUIPP app reduces women’s anxiety and/or helps her to come to decisions about her care that she is happy with. Women may have different preconceptions about the significance of symptoms of TPTL and varying perspectives on consequences of admission and or transfer, such as financial anxieties and separation from family, friends and other children [11]. Furthermore, the use of an app to guide clinical management is new to healthcare practice. Given the importance of shared decision-making emphasized by the Montgomery ruling [17], women’s views regarding its application in threatened preterm labour will be central to the future development of the QUIPP app.

## Methods/design

### Trial objectives

Our aim is to evaluate the ability of the QUIPP app to reduce inappropriate management of TPTL. This will externally validate the prediction model, assess its success as a clinical decision making tool, and measure its potential cost-saving impact in the emergency obstetric setting.

#### **Hypothesis:** The implementation of the QUIPP app and management algorithm will decrease inappropriate management for TPTL

### Trial design

EQUIPTT is a cluster randomised controlled trial (incorporating a parallel group design) across 13 obstetric centres. This design was chosen as for this scale of protocol intervention (in an emergency situation), it would be challenging to randomise at participant level, affecting uptake and generalisability of the findings, and individual clinicians cannot be randomised to varying decision-making with individual patients.

The pragmatic approach was taken to introduce the QUIPP app to entire hospital antenatal units, as standard practice for all clinicians, and all affected pregnancies. Using this parallel cluster studies have the added value of limiting the time-bias which occurs with a stepped wedge design. Also, unlike in a crossover design, the intervention does not need to be retracted from the cluster, avoiding contamination bias. Individuals within a cluster tend to have more similar outcomes than across clusters. The similarity in the outcomes of individuals within a cluster within a time period is typically measured by the within-cluster within-period intra-cluster correlation coefficient. If this is not factored into the power calculation and analysis, the effectiveness of the intervention may be exaggerated [18].

All 13 centres will provide data related to the principal outcome under their current practice in a six-week pre-intervention data collection period. Following randomisation, the centres will use intervention (using the QUiPP app) or control (routine management) for a nine-month analysis period. In the final phase of the trial, the intervention will be introduced in the 6 control units. Following this, data will be collected for a further six weeks across all 13 sites, in order to equally incentivise all sites to participate. The QUIPP app approach will be adopted as standard practice in all sites if successful. A list of the study sites is available from the authors on request.

An health economics analysis will also be conducted; this will involve a cost-minimisation and cost-consequences analysis of the use of the QUIPP app for triage and transfer compared to current practice. We hypothesise that there will be no negative impact on the health and wellbeing of women and neonates, as a result of the intervention, particularly given its non-invasive nature and that the intervention will be cost saving.

Women’s experience of TPTL assessment will be explored using self-completed questionnaires, with a subset of participants being invited to semi-structured interview.

### Sample size

For inappropriate admission decisions, a total sample size of 580, approximately 50 recruits per site, was calculated. Data from our group’s ongoing prospective observational study into the ability of qfFN to predict preterm birth in symptomatic women (PETRA REC Ref. 14/LO/1988) has allowed us to estimate the likely treatment effect for the intervention reducing inappropriate admissions from 25 to 10% and intra-class correlation 0.030. Based on 13 clusters this treatment effect requires approximately 580 for 80% power. We are aware that a larger number of centres would also be preferable, but believe 13 is adequate and at the limit of what is currently feasible.

For our qualitative questionnaires, we have estimated a target recruitment of 300 participants, 25 recruits per site, allowing for non-compliance and data collection errors. Based on previous research involving visual analogue scale (VAS) scores [19], a total sample size of 272 was required at standard levels of significance (alpha = 0.05 two-sided) to achieve 90% power to detect a 10% difference in mean VAS scores between hospitals with and without the intervention.

### Randomisation

Randomisation is at the cluster level. Centres have been randomly allocated to receive either intervention or control. Due to there being an odd number of centres, seven sites were allocated to one group and six to the other. Minimisation of the randomisation list was not appropriate for this design but the imbalances between three levels of NICU facility at intervention and control sites were small (4:3, 3:2, and 0:1). TPTL rates were known for 10 sites and the difference between the average rates at control and interventions sites were within chance (72 and 89%).

### Data analysis

#### Cluster randomised trial

Data analysis will follow the intention to treat principle, according to the planned intervention. Data will be analysed using Stata software Version 14 or later (StataCorp, College Station, Texas) to estimate the size and test for statistical significance of any effects of the intervention on primary and secondary, and analysis will be supervised by the trial statistician, Mr. Paul Seed. A per protocol analysis will also be performed, excluding those who are incidentally admitted or treated for reasons unrelated to preterm labour. Treatment effects for binary endpoints will be expressed as risk ratios (relative risk) with 95% CIs, using binomial regression and adjusting for variables used in the minimization process. Risk differences will also be calculated for the primary endpoint. The analysis model will include a random effect for clustering and adjusted standard errors for clustering. Adjustments will also need to be made for differences between cluster populations, such as ethnicity and maternal age. Our primary outcome of inappropriate decisions will be measured per 1000 deliveries.

#### Health economics evaluation

For the health economics analysis, a multi-level general linear model using appropriate family and link function, will be used to calculate the average cost per participant of use of the QUIPP app for triage and transfer compared to current practice. Costs will also be reported alongside secondary clinical outcomes as part of a cost-consequences analysis. We are not conducting a cost per quality adjusted life year (QALY) analysis (also called cost-utility analysis) given the hypothesis that the intervention is cost saving and does not result in any health decrement. These assumptions make a cost per QALY analysis redundant. Any health improvement will be captured by adverse incident reporting and secondary maternal and neonatal clinical outcomes, which will be reported alongside costs as part of the cost-consequences analysis.

#### Women’s experience study (EQUIPTT-Q)

When feasible (i.e. participants identified prior to assessment and staff available to obtain consent), participants in the cluster randomised trial will be invited to complete a questionnaire booklet. This includes questions about her symptoms, anxiety levels before and after her clinical assessment, the tests she received, her options for further management and the extent to which she is content with the decisions made. Anxiety levels and decisional conflict will be ascertained by visual analogue scale (VAS), and the Decisional Conflict Scale (DCS) questions [19]. The questionnaire booklet has been designed in collaboration with the Women’s Health Academic Centre’s Preterm Birth Studies public and patient involvement (PPI) panel. Difference in VAS anxiety scores between women before and after assessment, and between hospitals with and without QUIPP app in use at the time of assessment, will be calculated (paired and independent samples t-test). Difference between DCS scores after assessment, between hospitals with and without QUIPP app in use, will also be calculated (paired and independent samples t-tests as appropriate). If the DCS and VAS data is found to be not normally distributed, non-parametric equivalent tests will be used.

A subgroup of participants (approx. 20–30) will be invited to provide a more in-depth account of their experiences during one-to-one face-to-face or telephone interviews. The interview schedule will be determined following interim analysis of the data and free text box comments. The Framework approach [20] will be used to analyse the qualitative data obtained from the interviews. This method of qualitative data analysis is suitable for use with the anticipated findings from this study. Data will be analysed using NVivo software.

### Data collection

Local research staff will prospectively collect data on all available eligible episodes of threatened preterm labour.

Key data for all patients (at control and interventions sites)

- Hospital number
- Gestation at presentation
- Quantitative fetal fibronectin values (if available)
- Cervical length measurements (if available)
- Current NICU availability
- IUT attempt planned.
- Patient characteristics (age, parity, ethnicity, risk factors for preterm labour)
- Outcome data as outlined above (5.1 and 5.2).

Anonymised participant data will be transcribed on to the study database prepared for the trial by MedSciNet (Stockholm, Sweden).

Anonymous data will be stored on the Preterm Clinical Network (PCN) Database (REC Ref. 16/ES/0093). Identifiers (initials, date of birth, hospital and NHS number) required to ensure accurate outcome data collection will be stored on the separate, but linked, PCN Patient Details Database which is only accessible to local site users.

Women will be followed up until postnatal discharge. Neonates will be followed up to discharge or 28 days (whichever is sooner). Local research staff will monitor expected dates of delivery and ensure timely collection of pregnancy outcomes. Outcome data (medical and economic) will be collected by review of obstetric handheld/electronic notes. Outcome data for women who are transferred outside their local unit will be obtained by contacting the referral hospital. The Emergency Bed Service and Neonatal Transport Service are supportive of the study and can assist us in tracking patients who are not accommodated in their local hospital.

For the health economics analysis data will be collected from routine medical records on antenatal admissions, length of stay, outpatient appointments, day cases and scans for women identified as participants in the trial from the time of first being identified as part of the trial until discharge following delivery. Healthcare resource group codes for each event will be collected and costs from National Reference costs applied. We will conduct a time and motion study of clinician time and resources spent identifying a suitable transfer location on a sample of transfers. We will also conduct a micro costing of the cost of a transfer. We will also collect and report statistics and costs per patient for mode of delivery for women identified as participants in the trial and ex-utero transfers and NICU stays for their neonates (adjusting for non-singleton births). We will include the cost of the intervention which will include the cost of any training and the cost of clinician time associated with using the app. This information will be collected from the app, patient notes and a time and motion questionnaire administered to clinicians for a subset of patients.

For the women’s experience study (EQUIPTT-Q) participants, following consent, the researcher will enter the woman’s initials, hospital number, date of birth and date of signing consent against the next consecutive EQUIPTT-Q ID number on the EQUIPTT-Q register. This ID number will be entered on the consent form and alternative pages of the questionnaire booklet. The participant will be asked to complete part 1 (questions regarding baseline demographics, her symptoms and anxiety levels prior to assessment) straight away. She will be asked to keep the booklet with her and to complete part 2 (tests, planned interventions, anxiety post assessment and decisional conflict scale questions) after her clinical assessment. She will then hand the completed booklet back to staff before leaving the unit. The researcher checks the booklet for completion and enters data on EQUIPTT-Q datasheet (spreadsheet). The datasheet (which contains no identifiable data) is emailed to trial management team on a weekly basis. Data will be merged into the EQUIPTT-Q SPSS database which is separate from the main trial database.

Participants selected for interview will be contacted and given further verbal and written information. If willing, arrangements will be made at a time and place convenient to the participant and they will be asked to sign a consent form prior to the interview taking place. This may be via telephone or face-to-face. The interview schedules will be designed following interim analysis of the questionnaire data and free text comments, and in consultation with the preterm birth PPI panel. Interviews will be recorded on digital audio equipment and then transcribed and prepared for analysis.

At the end of the study and once outcomes have been collected by individual site users, the anonymous data will be extracted for analysis by HW from the PCN Database onto an Excel spreadsheet. The spreadsheet will be stored on a secure password protected computer for review by two researchers to remove duplicates and participants with missing data that should not be included in the analysis (HW and NC). Data cleaning will also be performed using Stata software Version 14 or later (StataCorp, College Station, Texas) by our trial statistician (PS) prior to analysis. The data extraction method will be piloted during the data analysis plan in the 9-month study period to ensure any potential pitfalls are identified.

### Data management

For the randomized cluster trial anonymised data only will be stored on a secure study-specific internet database (MedSciNet). All Investigators and study site staff must comply with the requirements of the Data Protection Act 1998 with regard to the collection, storage, processing and disclosure of personal information and will uphold the Act’s core principles.

Local principal investigators will ensure that the confidentiality of the patients’ data is preserved. As permitted by all applicable laws and regulations, limited participant attributes such as, age or date of birth may be used to verify the participant and accuracy of the participant’s unique identification number. Individual participant data will not be disclosed outside of study staff and will not appear on any reports produced by the sponsor. The following people may also access these records:

- Study monitors and auditors (including the data monitoring committee), who may work for the sponsor or its authorised representatives, who check that the study is being performed correctly and that the information collected is accurate.
- National and international regulatory authorities involved in keeping research safe for participants.

Participant information provided by the EQUIPTT-Q questionnaire will be labelled with a unique EQUIPTT-Q identification number. It will not include and patient-identifiable information. In order to arrange the interviews, the contact details (email, phone number) of willing participants will be obtained from hospital records only if the participant has been selected for interview.

Published results will not contain any personal data that could allow identification of individual participants.

A data monitoring committee (DMC), independent from the sponsor, will evaluate outcome data (e.g. adverse neonatal outcomes and maternal hospitalisation) in a blinded fashion at regular intervals through the trial with the ability to report any clinical concerns that may arise from the blinded data. Because of the parallel cluster design, data will only be available on the unmasked results at the end of the trial. Depending on the DMC’s requests, the closed DMC session will be able to consider the results of the primary maternal and neonatal endpoints in both arms of the trial and any Serious Unexpected Adverse Events and form a view on whether it is ethical for the trial to continue.

### Selection of participants

#### Main study inclusion criteria

- Pregnant women with symptoms of TPTL (contractions or abdominal pain)
- Between 23^+ 0^ and 34^+ 6^ weeks
- Capacity for quantitative assessment of fetal fibronectin and/or transvaginal ultrasonic cervical length if randomised to the intervention group.

#### Main study exclusion criteria

- Definitive diagnosis of labour (i.e. regular painful contractions with cervical change > 3 cm on speculum or digital examination)
- Confirmed ruptured membranes (on speculum examination)
- Significant vaginal bleeding

#### Women’s experience (EQUIPTT-Q) study inclusion criteria

- Pregnant women with symptoms of threatened preterm labour (contractions or abdominal pain)
- Between 23^+ 0^ and 34^+ 6^ weeks

#### Women’s experience (EQUIPTT-Q) exclusion criteria

- Unable or unwilling to give informed consent
- Under 16 years of age
- Unable to understand English language sufficiently to complete the questionnaire booklet

### Implementing the QUiPP app intervention

In each phase, each cluster will recruit consecutive women with singleton or twin pregnancies presenting between 23^+ 0^ and 34^+ 6^ weeks presenting with symptoms of preterm labour. Eligible participants will be identified at labour ward and day assessment triage by the direct care team and local research support teams. Inclusion and exclusion criteria are defined above.

All sites will receive training on the use of the QUIPP App and management guidance prior to its introduction, and periodically throughout the trial at peaks of staff turnover. This training will be delivered by the trial team at different times and locations to suit each site, for example, at departmental audit meetings and staff induction. Training will be delivered via clinical vignettes which provide opportunity for the clinicians to use the app in a training environment. Aide-memoires such as lanyards and pens are provided to each site to encourage use of the app. The doctor/midwife assessing the woman inputs the gestation, previous history of late miscarriage or preterm birth, quantitative fetal fibronectin value and/or ultrasonic cervical length into the app and the app provides % risk of delivery within 1/2/4 weeks and before 30/34/37 weeks. The exact thresholds for admission or treatment are gestation-dependent and may need to be tailored to individual circumstances. However as a recommendation, our guidance will suggest a 5% risk of delivery within 7 days as threshold for antenatal corticosteroid administration, admission, and/or in-utero transfer. Ultimately however the management decisions following the knowledge provided by the QUIPP app will remain the clinicians’ responsibility. A feedback option is available on the app to directly contact the trial team with specific non-clinical queries. Incentives and competitions will also aim to increase recruitment for the qualitative recruitment.

### Outcomes

#### Primary outcomes

Our primary outcome of inappropriate management for threatened preterm labour is defined by:

- number of inappropriate admission decisions: admitted and delivery interval > 7 days OR not admitted and delivery interval < 7 days

and

- number of inappropriate in-utero transfer decisions/actions: in-utero transfers that occurred or were attempted > 7 days prior to delivery, and ex-utero transfers within 24 h that should have been in-utero (attempted and non-attempted)

#### Secondary outcomes

Secondary outcomes will include:

- All components of primary outcome individually
- Maternal clinical outcomes (e.g. new onset gestational diabetes, thromboembolic disease and confirmed sepsis)
- Neonatal clinical outcomes (e.g. neonatal death prior to discharge, gestational age at delivery, birthweight, days of supplemental oxygen)
- Process measures (days of maternal hospitalisation, steroid, tocolytic and magnesium sulphate administration, neonatal intensive-care admissions, ex-utero transfer within 24 h of delivery)
- Compliance to management recommendations

Information will also be collected on the number of preterm deliveries before 34, and 37 weeks, and on the use of treatments to prevent prematurity [antibiotics, cerclage (by site), progesterone]. The authors may be contacted for samples of the data collection forms.

#### Health economic outcomes

Cost-savings will be generated as a result of:

- Reduction in the number of inappropriate admission decisions.
- Reduction in the number of inappropriate in-utero transfer decisions.

#### Women’s experience study (EQUIPTT-Q)

Our key outcome is difference in VAS anxiety scores between women before and after assessment, between hospitals with and without QUIPP app in use at the time of assessment. We will also analyse difference between DCS scores after assessment, in hospitals with and without QUIPP app in use.

## Discussion

Management of TPTL has represented a clinical conundrum for decades. Intervention “just-in-case” results in many unnecessary admissions, women being transferred out of their local hospital unnecessarily and most women receiving unnecessary drugs, such as steroids and tocolytics. It also prevents appropriate transfers as neonatal cots are blocked unnecessarily, resulting in more dangerous ex-utero transfers. One reason for the current trend for over-treatment of TPTL is concern that the false-negatives any triage system entails. This study will determine the risks and benefits of limiting management and treatment to those most at risk of delivery.

The exact thresholds for admission or treatment are gestation-dependent, may need to be tailored to individual circumstances and will remain the clinicians’ responsibility. As a recommendation, our guidance will suggest a 5% risk of delivery within seven days as threshold for antenatal corticosteroid administration, admission, and/or in-utero transfer. A 5% threshold is equivalent to an odds of 5:95 which is 1:19. This estimates the harm of a missed imminent delivery (a false negative) is 19 times as great as the harm of overtreatment (a false positive). It was found to be an acceptable threshold for intervention in our Delphi survey of preterm birth specialists. In the context of national guidance which advocated more intervention and disregarded the risk of harm this incurs to the majority [2], this study’s comprehensive evaluation of the true clinical impact of the QuiPP app will determine and justify an appropriate threshold for TPTL intervention for the first time.

If, as we hypothesise, the QUIPP app improves selection of the appropriate women for admission and transfer, it will ensure that therapies known to reduce risk of preterm neonatal morbidities are offered to those who need them and reduce the associated maternal and fetal risks to women who do not. Conversely if a treat-all policy is justified, we can help define resource use and clinical need to inform policy makers. Beyond obstetrics, evaluating the impact of an app in an emergency setting, and our emphasis on balancing harms of over-treatment as well as under-treatment, make EQUIPTT a valuable contribution to translational medicine.

## Abbreviations

CI: Chief investigator
DCS: Decision conflict score
EQUIPTT: The Evaluation of the QUIPP app for Triage and Transfer
EQUIPTT-Q: EQUIPTT Women’s Experiences Study
NHS R&D: National Health Service Research & Development
NPV: Negative predictive value
Participant: An individual who takes part in a clinical trial
PI: Principle Investigator
PPI: Patient and Public Involvement
PPV: Positive Predictive Value
QALY: Quality Adjusted Life Year
qfFN: Quantitative fetal fibronectin
REC: Research Ethics Committee
TPTL: Threatened Preterm Labour
TSC: Trial Steering Committee
VAS: Visual Analogue Scale
